# Group Theory Analysis to Study Phase Transitions of Quasi-2D Sr_3_Hf_2_O_7_

**DOI:** 10.3390/nano11040897

**Published:** 2021-03-31

**Authors:** Estelina Lora da Silva, Adeleh Mokhles Gerami, P. Neenu Lekshmi, Michel L. Marcondes, Lucy V. C. Assali, Helena M. Petrilli, Joao Guilherme Correia, Armandina M. L. Lopes, João P. Araújo

**Affiliations:** 1 IFIMUP, Institute of Physics for Advanced Materials, Nanotechnology and Photonics, Department of Physics and Astronomy, Faculty of Sciences, University of Porto, Rua do Campo Alegre 687, 4169-007 Porto, Portugal; neenulekshmi@fc.up.pt (P.N.L.); armandina.lopes@fc.up.pt (A.M.L.L.); jearaujo@fc.up.pt (J.P.A.); 2 School of Particles and Accelerators, Institute for Research in Fundamental Sciences (IPM), P.O. Box 19395-5531 Tehran, Iran; adeleh.mokhles.gerami@cern.ch; 3 CERN, Esplanade des Particules 1, 1211 Geneva 23, Switzerland; Guilherme.Correia@cern.ch; 4 Instituto de Física, Universidade de São Paulo, Rua do Matao 1371, São Paulo 05508-090, Brazil; michel@if.usp.br (M.L.M.); lassali@if.usp.br (L.V.C.A.); hmpetril@if.usp.br (H.M.P.); 5 C2TN, Departamento de Engenharia e Ciências Nucleares, Instituto Superior Técnico, Universidade de Lisboa, Estrada Nacional 10, 2695-066 Bobadela, Portugal

**Keywords:** new functional quasi-2D materials, energy harvesters, density functional theory, Ruddlesden Popper perovskites

## Abstract

We present an *ab-initio* study performed in the framework of density functional theory, group-subgroup symmetry analysis and lattice dynamics, to probe the octahedral distortions, which occur during the structural phase transitions of the quasi-2D layered perovskite Sr_3_Hf_2_O_7_ compound. Such a system is characterized by a high-temperature *I4/mmm* centrosymmetric structure and a ground-state *Cmc2_1_* ferroelectric phase. We have probed potential candidate polymorphs that may form the *I4/mmm* → *Cmc2_1_* transition pathways, namely *Fmm2*, *Ccce*, *Cmca* and *Cmcm*. We found that the band gap widths increase as the symmetry decreases, with the ground-state structure presenting the largest gap width (∼5.95 eV). By probing the Partial Density of States, we observe a direct relation regarding the tilts and rotations of the oxygen perovskite cages as the transition occurs; these show large variations mostly of the O *p*-states which contribute mostly to the valence band maximum. Moreover, by analyzing the hyperfine parameters, namely the Electric Field Gradients and asymmetric parameters, we observe variations as the transition occurs, from which it is possible to identify the most plausible intermediate phases. We have also computed the macroscopic polarization and confirm that the *Cmc2_1_* phase is ferroelectric with a value of spontaneous polarization of 0.0478 C/m^2^. The ferroelectricity of the ground-state *Cmc2_1_* system arises due to a second order parameter related to the coupling of the rotation and tilts of the O perovskite cages together with the Sr displacements.

## 1. Introduction

Ruddlesden Popper (RP) layered perovskites are formed by a rock-salt layer positioned between perovskite (ABO_3_)n blocks—(AO)(ABO${}_{3}$)n, either within double-layered compounds—A${}_{3}$B${}_{2}$O${}_{7}$ [1,2,3] or in A-site ordered single-layered compounds—AA’BO${}_{4}$ [4,5]. These quasi-2D structures present advantages of possessing similar properties to that of conventional 2D materials [6], without the need to scale down the system to the atomic-layer thickness. Such properties include quantum confinement effects which lead to large exciton binding energies, high quantum yields and other photophysics properties, making these systems also desirable candidates for next-generation energy-efficient optoelectronics [6,7].

RP systems have another advantage of being able to also evidence switchable electric polarization (ferroelectric materials) thus being attractive compounds due to their technological importance for nonvolatile memories, sensors, actuators, etc. [8,9]. Noncentrosymmetric polar perovskites may exhibit proper ferroelectricity (i.e., ABO${}_{3}$) which arises due to the center-of-symmetry breaking of the A-site and/or B-site cations [10]. On the other hand, improper ferroelectrics evidence polarization due to geometrical structural constraints which are primarily associated with the perovskite oxygen polyhedral distortions [11]. Hybrid improper ferroelectricity (HIF) is yet another phenomenon of spontaneous electric polarization arising from antiferrodistortive displacements linked to rotations and tilts of the perovskite octahedra [12,13]. The polarization is thus induced by the coupling of two nonpolar lattice modes, i.e., octahedral rotations and/or tilts, for which these two modes can condense at the same temperature (an avalanche transition) or at different temperatures (a staggered transition) [14]. For improper ferroelectric systems, the two distortion patterns will only evidence an avalanche transition [13]. HIF materials express their significant potential in the design of novel materials with variable functions ranging from superconductors, multiferroics, to materials that display negative thermal expansion [4]. Besides this, recent findings indicate the importance of HIF materials in the development of reliable and nonvolatile memory due to the unique properties, including the switching of spontaneous polarization with an applied external electric field [15]. HIFs tend to exhibit very large temperature-independent dielectric constants, with practical interests for technological applications, such as for alternative dielectrics to silicon dioxide for memory and logic devices. This property is distinguishable from proper ferroelectrics, for which the dielectric constant is typically large around the phase transition temperature and also differs from that of improper ferroelectrics that exhibit a temperature-independent however small dielectric constant [12].

In the case of double-layered RP structures (i.e., A${}_{3}$B${}_{2}$O${}_{7}$), HIF is induced due to the coupling of the nonpolar octahedral rotation (condensation of in-phase rotation and out-of-phase tilting modes) with a ferroelectric polar mode (cation displacements) [3,16,17]. Thus, the interdependence of polarization to other physical properties is expected in these compounds, for example, electric-control of magnetization function [13].

The first two double-layered RP candidates that have been theoretically investigated by Benedek and Fennie [13] were Ca${}_{3}$Ti${}_{2}$O${}_{7}$ (CTO) and Ca${}_{3}$Mn${}_{2}$O${}_{7}$ (CMO) and which are derived from the parent perovskite CaTiO${}_{3}$ and CaMnO${}_{3}$, respectively. The authors report that the values of the macroscopic polarization for Ca${}_{3}$Ti${}_{2}$O${}_{7}$ of 20 µC/cm${}^{2}$ and Ca${}_{3}$Mn${}_{2}$O${}_{7}$ of 5 µC/cm${}^{2}$ [13]. Oh et al. [1] have experimentally observed ferroelectricity in Ca${}_{3}$Ti${}_{2}$O${}_{7}$; however, the reported polarization (∼10 µC/cm${}^{2}$) is somewhat lower than that of the theoretical value. At high temperature, the parent perovskites of both CTO and CMO crystallize in a nonpolar high-symmetry *I4/mmm* (S. G > 139) phase, and at room temperature the CTO as well as CMO lower the symmetry towards a non-centrosymmetric polar *Cmc2${}_{1}$* (S.G. 36) ground-state phase. The *Cmc2${}_{1}$* symmetry is represented by the Glazer tilt notation (aac${}^{+}$) wherein out-of-phase oxygen octahedral tilt about [110], and in-phase oxygen octahedral rotations about [001] are allowed [18]. Experimentally, it was seen that CMO exhibits a structural transition path from low-temperature *Cmc2${}_{1}$*, intermediate *Ccce* (S.G. 68) and high-temperature *I4/mmm* (S.G. 139) [18].

Recently, ferroelectric *Cmc2${}_{1}$* phases have also been observed in Sr-compounds such as Sr${}_{3}$Sn${}_{2}$O${}_{7}$ [19] and Sr${}_{3}$Zr${}_{2}$O${}_{7}$ [20]. This opened up the possibility of probing further Sr-based compounds, such as Sr${}_{3}$Hf${}_{2}$O${}_{7}$ (SHO) that has been theoretically studied through first-principles [21]. The authors probe potential ferroelectric switching paths between the *I4/mmm* and *Cmc2${}_{1}$* phases by employing the nudge elastic band (NEB) method. From respective work it was found that a four-step pathway across the nonpolar *Pnma* (S.G. 62) and *Pbcn* (S.G. 60) phases were the most energetically favourable barriers for SHO [21]. In addition, Benedek and Fennie [13,22] have indicated that for the CMO system, the phase transition should proceed through an intermediate phase either by passing through the *Cmcm* or the *Cmca* phase [13,22]. The research groups of Liu et al. [23] and Rodrigues et al. [18] have experimentally observed the *Cmcm* and *Ccca* phases, respectively, as being intermediate structures that constitute the pathway between the high-temperature and ground-state systems of the CMO compound. Moreover, with respect to the Sr-based systems, experimental studies by Yoshida et al. [2,20] confirmed the existence of an intermediate nonpolar *Cmcm* phase for Sr${}_{3}$Zr${}_{2}$O${}_{7}$ and *Ccce* for Sr${}_{3}$Sn${}_{2}$O${}_{7}$ along the structural transition from *I4/mmm* to *Cmc2${}_{1}$*. Moreover, it is found that for both the Sr${}_{3}$Zr${}_{2}$O${}_{7}$ and the Sr${}_{3}$Sn${}_{2}$O${}_{7}$ systems an antipolar phase, the *Pbcn* (S.G.60), competes with the equilibrium polar *Cmc2${}_{1}$* phase [20]. On the other hand, Wang et al. [19] predicts that it is the *Cmca* phase which will result from the orthorhombic structural distortions together with *Cmc2${}_{1}$*, therefore resulting in orthorhombic twins.

We intend to demonstrate from the present work that by employing first-principles calculations on SHO, it is possible to predict possible transition pathways based on group-subgroup relations, as shown by Benedek and Fennie [13,15] on the CMO systems. We show an energetic stability trend of the potential intermediate phases between the *I4/mmm* towards the *Cmc2${}_{1}$* system, with the electronic energy lowering as the symmetry decreases. On the other hand, we observe an opposite trend of the electronic band gaps, since the widths increase as the symmetry of the structure decreases. By analyzing the lattice dynamics of all the potential systems we observe imaginary modes for all the phases (consistent of being transition states for the given conditions). Exception is observed for the *Cmc2${}_{1}$* polymorph, which evidences dynamical stability and therefore results confirm that *Cmc2${}_{1}$* is in fact the ground-state system for SHO. By computing the spontaneous polarization, we show that the *Cmc2${}_{1}$* phase is ferroelectric; these results are consistent to what has been reported in literature for the same family of compounds, i.e., CMO, CTO [3,13,18] and also for SHO [21].

Nuclear hyperfine techniques are particularly important to study local magnetic and electric interactions, such as the electric field gradient (EFG), at a given nuclear site. The measurement of the EFG, through perturbed angular correlations (PAC) experiments, combined with *ab-initio* density functional theory (DFT) calculations, has shown to be a valuable tool to probe the octahedral rotations of the cages during phase transition [18]. Therefore, we have computed the EFG for each structural phase and observe variations of respective parameters which allow the possibility to relate the intermediate phases with the most plausible structural transition pathway.

## 2. Materials and Methods

The calculations have been performed within the framework of the density functional theory (DFT) [24,25] in the Kohn–Sham scheme, as implemented in the Quantum Espresso (QE) code [26,27,28] and in WIEN2k [29]. The exchange-correlation functional was described by the semi-local generalized-gradient approximation with the Perdew–Burke–Ernzerhof parametrization revised for solids (PBEsol) [30,31].

Structural relaxations and lattice dynamics calculations were carried out with the Projector Augmented Wave (PAW) method [32] using the setups: Sr[4s${}^{2}$4p${}^{6}$5s${}^{2}$], Hf[5s${}^{2}$5p${}^{6}$5d${}^{2}$6s${}^{2}$] and O[2s${}^{2}$2p${}^{4}$]. The starting point for our calculations was fully structural relaxations of the different phases, using the variable cell-shape relaxation (by damped Beeman ionic dynamics and the Wentzcovitch extended Lagrangian for the cell dynamics [33]) performed with 70 Ry plane wave expansion energy cut-off and 12 × 12 × 6 **k**-point Monkhorst-Pack mesh [34].

The theoretical background regarding the harmonic lattice-dynamics calculations is detailed in References [35,36]. Lattice-dynamics calculations were performed using the supercell finite-displacement method implemented in the Phonopy software package [37], with QE used as the 2nd order force-constant calculator. Calculations to obtain the phonon dispersion curves were carried out on 2 × 2 × 2 expansions of the primitive-cell.

**Figure 1:**
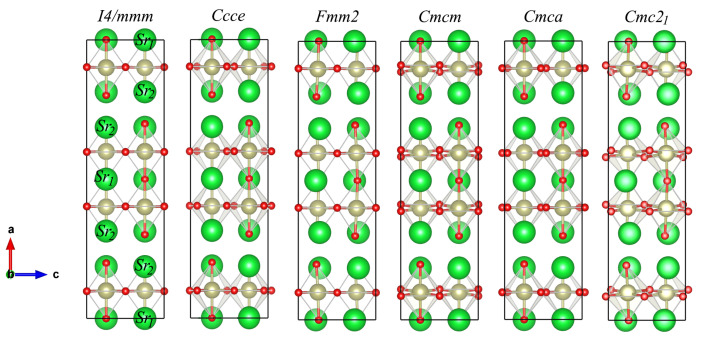
Representation of the different structural phases of Sr${}_{3}$Hf${}_{2}$O${}_{7}$, namely the parent *I4/mmm* structure, followed by the *Ccce*, *Fmm2*, *Cmcm*, *Cmca* and the ground-state *Cmc2${}_{1}$* structural phases. The Sr${}_{1}$ and the Sr${}_{2}$ located at the A’ and at the A sites, respectively, are labeled accordingly on the *I4/mmm* phase, whose label can be extrapolated to the remaining phases. O ions are shown in red, Sr in green and Hf in gold. The figures were plotted using the VESTA visualization software [48].

The Full Potential Linearized Augmented Plane Wave (FP-LAPW) method as implemented in the WIEN2k code [29] was employed to obtain the hyperfine parameters including electric field gradients at the nuclear sites (EFG) (largest diagonal component in the principal axis system, V${}_{zz}$, and the asymmetry parameter, η), have been calculated for the different phases of SHO, namely *I4/mmm*, *Cmcm*, *Cmca*, *Ccce*, *Fmm2*, *Cmc2${}_{1}$* (structures represented in Figure 1). As an all-electron method, WIEN2k has proven to be a benchmark to compute hyperfine parameters; the macroscopic polarization, by employing the Berry’s phase expressions [38,39,40], have also been computed with the WIEN2k code and for the above mentioned structural phases. The Sr, Hf, and O radii of the muffin-tin atomic spheres were respectively set to 2.25, 2.07, and 1.78 a.u., the energy boundary between core and valence electronic states was -6 Ry, the cut-off parameter RMT × KMAX (which controls the size of the basis set) was 7.0 and G${}_{\max}$, the Fourier expansion of the charge density, was restricted to 16 Ry${}^{1/2}$. The geometry optimizations of different phases were obtained through the total energy minimization with respect to the unit cell volume using the 3rd order Birch–Murnaghan equation of state (EoS) [41,42], and internal atomic positions were minimized to a force limit below 1 mRy/a.u. A mesh of (6 × 6 × 6) **k**-points in the irreducible part of the first BZ was applied to the self-consistent total energy calculation. We have also employed the modified Becke-Johnson (mBJ) exchange-correlation functional [43] to compute the band gap energies. The mBJ functional provides an efficient framework for band gap prediction and is an appealing alternative to hybrid functionals and the many-body perturbation *GW* method for large systems and has been successfully applied to different perovskites structures [44,45,46,47].

The calculation of the EFG tensor can provide the signature of observable pathways on the structural phase transition from the *I4/mmm* system towards the ground-state *Cmc2${}_{1}$* structure. The EFG tensor components are defined as the second order spatial derivatives of the self-consistent potential *V* evaluated at a given nuclear site [49]

(1) $$V_{\mathrm{ij}}=\frac{\partial^{2}V}{\partial_{i}\partial_{j}},$$

where i=x,y,z. Since $V_{\mathrm{ij}}$ is a symmetric (traceless) second rank tensor, it can be diagonalized (the principal system of axis) and the largest diagonal component $V_{\mathrm{zz}}$ is commonly referred as the EFG and defined such that $|V_{\mathrm{zz}}\left|⩾\right|V_{\mathrm{yy}}\left|⩾\right|V_{\mathrm{xx}}|$.

Due to the traceless property, only two parameters are enough to characterize the EFG tensor and these are usually chosen as $V_{\mathrm{zz}}$ and the asymmetry parameter

(2) $$\eta =\frac{V_{\mathrm{xx}}-V\mathrm{yy}}{V_{\mathrm{zz}}}.$$

## 3. Results

The general formula of SHO can be written as (AHfO${}_{3}$)${}_{2}$A’O with A’ = Sr${}_{1}$ and A = Sr${}_{2}$. The environment around the two Sr atoms are inequivalent. As shown in Figure 1, different structures are formed by rock-salt (R) and perovskite (P) blocks which include the Sr${}_{2}$ located at the A site and the Sr${}_{1}$ on the A’ site, respectively. The P block consists of two layers of corner-sharing HfO${}_{6}$ octahedra along the c-axis. While the A’-site cations occupy a twelve-fold coordination environment (cuboctahedral), the A cation is at the nine-coordination site. As observed in Figure 1, the *I4/mmm* structure exhibits higher symmetry being the paraelectric high-temperature phase of the system, analogously to other RP oxides, such as Ca${}_{3}$Ti${}_{2}$O${}_{7}$ and Ca${}_{3}$Mn${}_{2}$O${}_{7}$ [13]. By decreasing the temperature, lower symmetry structural phases may be generated by inducing tiltings and/or rotations of the O octahedral cages, namely the *Ccce*, *Fmm2*, *Cmcm*, *Cmca*, and *Cmc2${}_{1}$* structures. Different phases of SHO, namely *I4/mmm* (S.G. 139), *Cmcm* (S.G. 63), *Ccce* (S.G. 68), *Cmca* (S.G. 64), *Fmm2* (S.G. 42), *Cmc2${}_{1}$* (S.G. 36) have been here simulated within the Kohn–Sham scheme of the DFT. The equilibrium lattice constants of the different phases calculated using the PBEsol functional with both WIEN2k and QE codes are shown in Table 1. Small differences, in the usual range (3–4% in volume), can be observed due to the different methods used in these codes.

### 3.1. Symmetry Mode Analysis

As mentioned above regarding the effect of the temperature lowering, most of the low-symmetry perovskite phases can be derived from the high-symmetry structure by rigid rotations and tiltings of the octahedral units around respective symmetry axes. The structural distortions relating the two phases can be denoted as a symmetry-breaking distortion [50,51]. The equilibrium position of the cation can therefore be determined by the positions and rotations/tiltings of the anions forming the octahedral cage for a given set of bond angles. By employing symmetry-mode analysis, it is possible to fully describe the distorted ground-state *Cmc2${}_{1}$* structure (S.G. 36) by decomposing the structural distortion into contributions from lattice modes with different symmetries. These are characterized by the irreducible representations (IR) of the centrosymmetric tetragonal *I4/mmm* (S.G. 139) structure. The analysis is performed using the software package AMPLIMODES [52] of the Bilbao Crystallographic Server, being useful to determine the driving mechanisms of the structural phase transitions and the fundamental instabilities at the origin of the distorted phases. By providing the high- and low- symmetry structures, the use of AMPLIMODES [52] allows to perform atomic mappings (pairings) by identifying which atoms in the low symmetry structure correspond to the asymmetry unit of the reference structure and therefore computing the atomic displacements, which relate both structures. From this approach, it is then possible to define a basis of symmetry-adapted modes and calculate the amplitudes and polarization vectors from the high-symmetry to the distorted structure [50,51,53].

Table 2 presents a summary of the basis of symmetry modes and respective IR which describe the atomic displacements in each Wyckoff orbit of the high symmetry phase. There are a total number of nineteen basis modes involving the displacements of the different atomic sites in the system. Eleven modes correspond to displacements of the three different O sites, namely with two referring to the sites at the 2a WP ($\Gamma_{5}^{-}$ and $X_{3}^{-}$), six to 8g ($\Gamma_{1}^{+}$, $\Gamma_{5}^{-}$, $X_{2}^{+}$, $X_{3}^{-}$) and three to the 4e WP ($\Gamma_{1}^{+}$, $\Gamma_{5}^{-}$ and $X_{3}^{-}$). Three modes refer to displacements of the Hf sites, namely $\Gamma_{1}^{+}$, $\Gamma_{5}^{-}$ and $X_{3}^{-}$. Regarding the Sr sites, two modes are related to the Sr${}_{1}$ displacements ($\Gamma_{5}^{-}$ and $X_{3}^{-}$) and three to the Sr${}_{2}$ site ($\Gamma_{1}^{+}$, $\Gamma_{5}^{-}$ and $X_{3}^{-}$). The atomic displacements of all atoms, multiplied by a given amplitude, yields the actual distortion of the low-symmetry structure. All O cages of the low-symmetry ferroelectric phase are rotated with respect to the *I4/mmm* phase; these rotations force the Sr atoms to deviate from the centrosymmetric positions, resulting in the antiferroelectric displacement of Sr atoms at the A-site. Noncanceled displacements then induce ferroelectric polarization.

**Figure 2:**
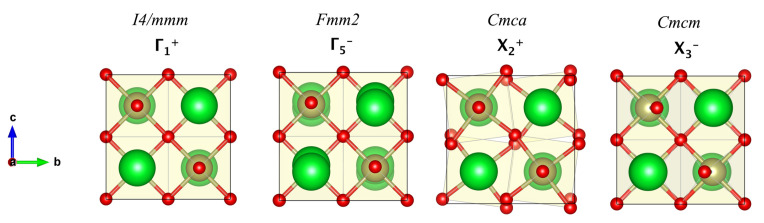
Decomposition of the structural distortion from *I4/mmm* → *Cmc2${}_{1}$* into contributions from lattice modes with different symmetries. The distorted structure derives from the high-symmetry structure through four frozen distortions, $\Gamma_{1}^{+}$, $\Gamma_{5}^{-}$, $X_{2}^{+}$ and $X_{3}^{-}$. Oxygen ions are shown in red, Sr in green and Hf in gold. The figures were plotted by using the VESTA visualization software [48].

Table 3 summarizes the mode decomposition, which lists the IR involved in the *I4/mmm* → *Cmc2${}_{1}$* distortion and the absolute amplitudes of the symmetry components of the global distortion. By analyzing Table 3, it can be observed that the modes with highest amplitudes are $X_{2}^{+}$ and $X_{3}^{-}$, with values of Q${}_{X_{2}^{+}}=0.5487$ Å and Q${}_{X_{3}^{-}}=0.7838$ Å, respectively, indicating that these modes are the major contributors to the distortion towards the low symmetry phase. By analyzing Figure 2, it is possible to deduce that the $X_{2}^{+}$ and $X_{3}^{-}$ IR correspond to in-plane rotations and the tilting of the perovskite O cages (HfO${}_{6}$), respectively, whereas IR $\Gamma_{5}^{-}$ corresponds mostly to the ferroelectric displacement of the Sr ions. It is noteworthy to mention that based on Table 2, we can verify that the $\Gamma_{5}^{-}$ mode also corresponds to displacements of the O atoms; however, these are basically negligible in comparison to the Sr movement, as can be observed in Figure 2. The IR modes obtained from respective analysis is compatible with the global structural distortion between the high- and low-symmetry phases relating both phases and these are compatible with symmetry-breakings observed in the same family of systems [3,13,15,18].

**Figure 3:**
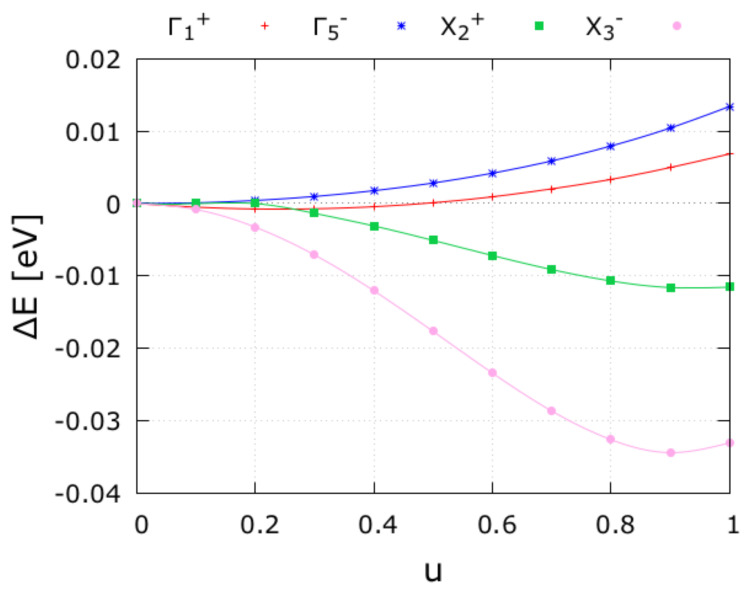
Potential energy surface (per formula unit) along each distortion mode, with u=0 being the centrosymmetric tetragonal *I4/mmm* structure and u=1 the distortion structure corresponding to the isotropy subgroup of the frozen mode. All four distortion modes contribute to the *Cmc2${}_{1}$* distortion, although most significant are the $X_{2}^{+}$ and $X_{3}^{-}$.

**Figure 4:**
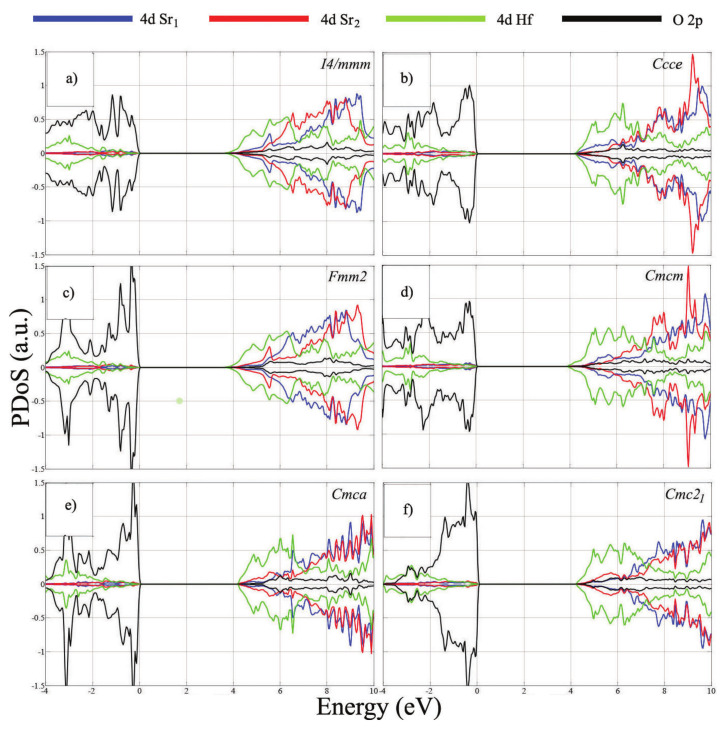
Partial density of states of the potential structural phases of SHO using the PBEsol functional: (**a**) *I4/mmm*, (**b**) *Ccce*, (**c**) *Fmm2*, (**d**) *Cmcm*, (**e**) *Cmca* and (**f**) *Cmc2${}_{1}$*. The VBM is aligned at the zero-energy level.

Figure 3 presents the potential energy surface along each distortion mode (frozen mode). The end-point u=0 corresponds to the high-symmetry *I4/mmm* structure, and u=1 represents the distortion corresponding to the isotropy subgroup of the respective frozen mode. The different frozen-mode distortions would thus correspond to the subgroups referenced in Table 3: $\Gamma_{1}^{+}$ distortion to the symmetry breaking towards the *I4/mmm* symmetry; $\Gamma_{5}^{-}$ distortion would correspond to the symmetry breaking towards the *Fmm2* phase; and $X_{2}^{+}$ and $X_{3}^{-}$ distortions would correspond to the symmetry breaking towards the *Cmca* and *Cmcm* symmetry, respectively. The sum of the four different u=1 end-points will agree with the ground-state *Cmc2${}_{1}$* symmetry. It can be observed that the energy lowers considerably along the $X_{2}^{+}$ and $X_{3}^{-}$ modes, which means that these distortions are mainly responsible for the symmetry-lowering to the *Cmc2${}_{1}$* phase. This corresponds to what has been discussed above (Table 3) and to these modes contributing with more weight to the distortion (Q${}_{X_{2}^{+}}=0.5487$ Å and Q${}_{X_{3}^{-}}=0.7838$ Å). These two modes are mostly due to the O cages, which evidence in-plane rotations and tiltings, respectively. This feature is consistent with what is observed from the electronic PDoS (Figure 4), for which the O-states are those which present major variations for the different studied structural phases. The $\Gamma_{1}^{+}$ will be omitted from the discussion since the amplitude distortion is practically negligible when compared to the other distortion amplitudes. The $\Gamma_{5}^{-}$ mode refers to motions of the Sr ions and produces an increase in energy when the frozen distortion occurs. In combination with other distortions that occur for the surrounding cage, the Sr movement leads to an average reduction in the overall energy of the SHO system.

In the following sections, we also include discussions of the analysis performed for another structural phase, i.e., *Ccce* (S.G. 68), since this is an experimentally known phase of the CMO system. Through space group relations, the *Ccce* phase is obtained through the out-of-plane rotations of the O cages, which corresponds to the condensation of a zone boundary $X_{1}^{-}$ [18].

### 3.2. The Relative Electronic Energy

The enthalpy of formation of a crystal can be calculated by using the following expression:

(3) $$\Delta H_{\mathrm{SHO}}=E_{\mathrm{SHO}}-3\mu_{\mathrm{Sr}}-2\mu_{\mathrm{Hf}}-7\mu_{O},$$

where $E_{\mathrm{SHO}}$ is the total electronic energy (obtained from DFT calculations) and $\mu_{\mathrm{Sr}}$, $\mu_{\mathrm{Hf}}$ and $\mu_{O}$ are the chemical potentials of the constituent Sr, Hf and O elements, respectively. We must note that when comparing the enthalpies of formation among the different phases with the same composition, the chemical potentials cancel out, and therefore only the $E_{\mathrm{SHO}}$ term survives.

We have computed the total energies of the representative structures that constitute the pathway between the paraelectric reference *I4/mmm* phase and the ferroelectric ground-states *Cmc2${}_{1}$* structure (Table 4). From Table 4 we observe that the polar *Cmc2${}_{1}$* phase presents the lowest energy, which is in good agreement with Ref. [21], establishing that the SHO ground-state is therefore ferroelectric. The system with higher energy is the tetragonal *I4/mmm*, followed by the *Ccce*, *Fmm2*, *Cmcm*, and *Cmca*. As mentioned before, the *Ccce* phase does not constitute the pathway between *I4/mmm* → *Cmc2${}_{1}$* according to group theoretical analysis; however, for the sake of completeness we have computed the structural parameters for this structure as well. From this analysis, we concluded that the energy ordering agrees with the discussion performed in Section 3.1, for which the most plausible transition pathway from the high- to the low-symmetry phase would be: *I4/mmm* < *Fmm2* < *Cmcm* < *Cmca* < *Cmc2${}_{1}$*. The energy of the *Ccce* phase is higher than that of the *Fmm2* system. However, to induce the *Ccce* structure the condensation of the $X_{1}^{-}$ mode would be required, which, and according to group theory analysis, does not occur naturally for the system. However, such a phase has been experimentally observed in Ca${}_{3}$Mn${}_{2}$O${}_{7}$ [18], which raises the possibility of occurrence of the *Ccce* phase also in SHO but as a first-order phase transition. Moreover, it has been observed that the intermediate *Ccce* structural phase may also occur in the Sr${}_{3}$Sn${}_{2}$O${}_{7}$ system [2], therefore providing further foundations for the theoretically investigation of this structural polymorph.

### 3.3. Partial Density of States

In order to investigate the effect of octahedral tilts/rotations on the electronic properties of SHO along the plausible path of the phase transition, the partial density of states (PDoS) have been computed (Figure 4). As observed in Figure 4, the conduction band minimum (CBM) is mostly defined by the Hf *d*-states, whereas the fully occupied O *p*-states form the valence band maximum (VBM). There is considerable hybridization at the higher energy levels of the conduction band, among the different Hf and Sr d-states. The overall PDoS features of the studied SHO structural phases are similar and all evidence an insulator behavior. Major differences are observed for the O p-states, which can be related to rotations of the octahedral cages, as mentioned in Section 3.1, and evidence largest contribution to the symmetry-breaking in the path towards the ground-state.

Moreover, the *Ccce* and *Cmca* structures evidence differences at the CBM. While for the remaining phases the CBM is mostly defined by the Hf *d*-states, these two mentioned *Ccce* and *Cmca* structures show hybridized Hf and Sr *d*-states and O *p*-states at the CBM. These features evidence dissimilarities with the remaining phases, probably caused by the out-of-plane and in-plane rotations of the octahedral cages (Sr *d*-states and O *p*-states), respectively, which lead to variations of the Hf *d*-states.

The width of the energy band gaps by employing the PBEsol and mBJ exchange-correlation functionals are summarized in Table 5. The mBJ approximation provides higher values than the PBEsol functional, as expected, due to the nature of the meta-GGA functional. To the best of our knowledge no experimental results regarding the band gaps of SHO have been reported, nevertheless the presently obtained PBEsol gap width for the *Cmc2${}_{1}$* phase is in good agreement with the 4.2 eV value obtained in Reference [21]. We must also note that the gap we have obtained is larger than in other RP systems, namely, for the heavier element system Ba${}_{3}$Ce${}_{2}$O${}_{7}$ [54] (∼2.4 eV using the PBEsol functional) and the Ca${}_{3}$Mn${}_{2}$O${}_{7}$ (∼0.8 eV using the PBE).

We note that the higher symmetry structures present lowest gap widths, a feature observed for both employed functionals (Table 5). This may be due to the fact that the top of the valence band is primarily formed by O-p states which, and as can be evidenced from Figure 4, exhibit a larger splitting in the lower symmetry systems. such a feature is consistent with the changes occurring in the O perovskite cages.

### 3.4. Phonon Dispersion Curves

We have performed lattice dynamics calculations in order to analyse the dynamical (in)stability of the different studied phases of SHO.

**Figure 5:**
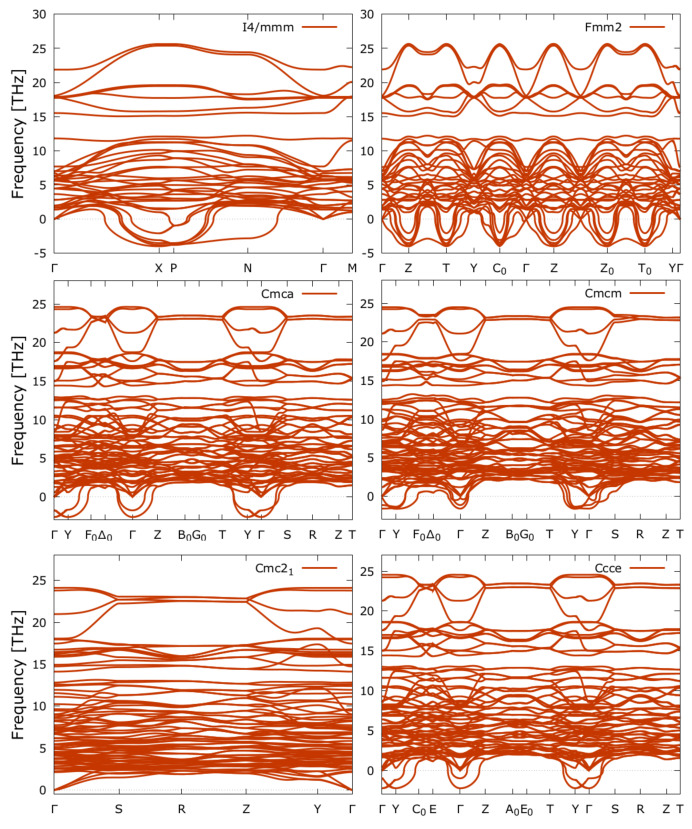
Phonon dispersion curves of the different structural phases that form the pathway between the high-symmetry *I4/mmm* phase and ground-state *Cmc2${}_{1}$* and including the *Ccce* structural phase.

By inspecting Figure 5, we can observe that the *I4/mmm* phase shows imaginary modes (represented by negative phonon frequencies) at the zone boundaries, more specifically at the high-symmetry points **X**, **P** and **N**.

The negative modes at the **X**-point are consistent with the symmetry mode analysis and related to the rotations and tilts of the O cages, namely the $X_{2}^{+}$ and the $X_{3}^{-}$ modes. As the distortion lowers the symmetry to *Fmm2*, the negative modes delocalize throughout the whole BZ (with exception of the **Y**- and Γ-points), characterizing the respective space group as being unstable, and thus a transition state to yet another phase.

According to Table 3, *Fmm2* is related to a mode that transforms as $\Gamma_{5}^{-}$ IR and this mode is shown to be stable in the phonon dispersion of the *I4/mmm* phase. The phonon dispersion further shows that these antidisplacements of the Sr atoms do not occur alone, since they are delocalized in the whole BZ. The results suggest that the octahedral instabilities drive the transition from the *I4/mmm* either to the *Cmcm* or *Cmca* phase, where imaginary modes occur at the **Y**- and Γ-points. We must note that the *Cmca* system shows quite localized imaginary frequencies located at the high-symmetry points of **Y** and the zone-center Γ. Interestingly enough the *Ccce* shows similarities in the phonon dispersions when compared to the *Cmca* system, with negative phonon branches located at the high-symmetry **Y**- and Γ-points. The distortions related to the tilting of the octahedral cages show similar phonon branches, with negative modes also localized at **Y**- and Γ-points. The only phonon dispersion spectra for which we cannot find any imaginary frequencies is for the *Cmc2${}_{1}$* phase, indicating that this phase is dynamically stable at 0 K for SHO, and in accordance to that obtained in Reference [21]. Moreover, as explained in Reference [13], the coupling between the oxygen octahedron rotation mode ($X_{2}^{+}$) and an oxygen octahedron tilt mode ($X_{3}^{-}$) establishes the polar *Cmc2${}_{1}$* space group, and for which a zone-center polar instability is not required ($\Gamma_{5}^{-}$).

### 3.5. Spontaneous Polarization

The spontaneous polarization of SHO for the different phases (end-points and intermediate) have been calculated using the Berry phase approach. The macroscopic polarization resulted in finite values for the *Fmm2* and *Cmc2${}_{1}$* phases, with P = 0.319 C/m${}^{2}$ and P = 0.0478 C/m${}^{2}$, respectively. The latter value is consistent with that computed in Reference [21], where it has been found that P = 0.043 C/m${}^{2}$. The value of *Fmm2* is quite large, which is expected since the movement of the Sr ions out of the equilibrium positions generate polarization. Since the $\Gamma_{5}^{-}$ mode is polar, proper ferroelectricity is induced from the *I4/mmm* to the *Fmm2* phase. However, and based on the phonon dispersion curves (mentioned above in Section 3.4), this transition would probably not occur since the system is dynamically unstable, evidencing negative frequencies throughout the whole BZ. For the *I4/mmm*, *Cmcm*, *Ccce*, and *Cmca* phases the polarization is null, which is consistent with the antiferrodistortive displacements of the O perovskite cages and also since these structures are centrosymmetric the polarization must be zero. We must also note that the value of polarization of the *Cmc2${}_{1}$* phase is comparable with those found for other compounds, such as of Sr${}_{3}$Sn${}_{2}$O${}_{7}$ with P = 0.039 C/m${}^{2}$ and Sr${}_{3}$Zr${}_{2}$O${}_{7}$ with P = 0.072 C/m${}^{2}$ [4]. The polarization of the Sr${}_{3}$B${}_{2}$O${}_{7}$ (B = Hf, Sn, Zr) compounds are found to be lower than the value of the Ca${}_{3}$Ti${}_{2}$O${}_{7}$ (1.0 C/m${}^{2}$ [1]) and Ca${}_{3}$Mn${}_{2}$O${}_{7}$ (0.5 C/m${}^{2}$) [13] systems, what can be related to the restricted atomic displacement of Sr (atomic radii: 215 pm) from the ideal position when compared to that of Ca (atomic radii: 197 pm), due to the cations radii size.

### 3.6. The Hyperfine Parameters of SHO

**Figure 6:**
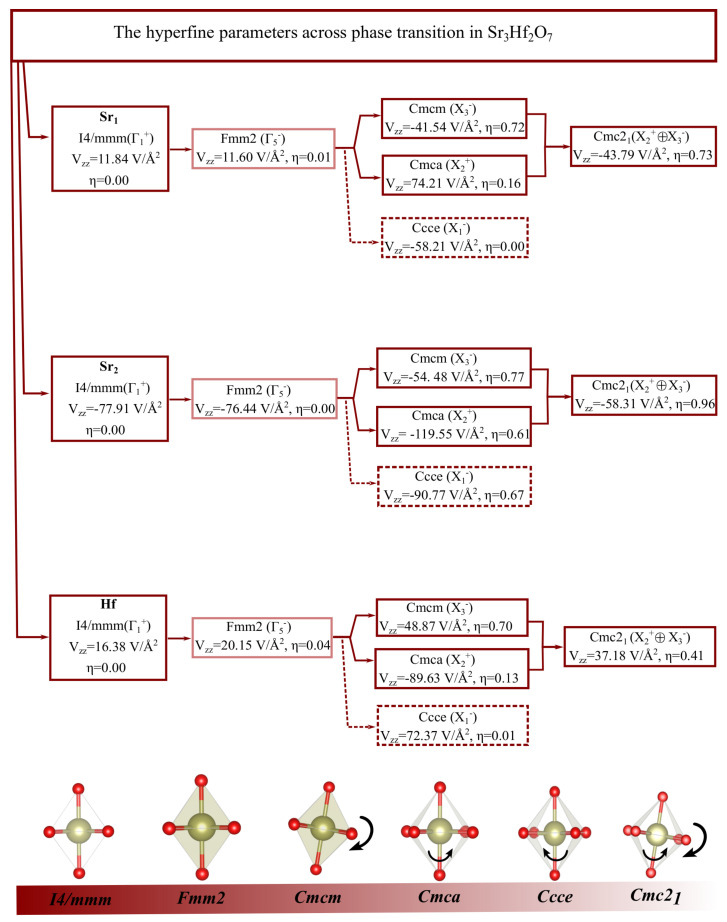
Hyperfine parameters obtained within DFT for the perovskite (Sr${}_{1}$), the rock-salt (Sr${}_{2}$) and Hf sites of Sr${}_{3}$Hf${}_{2}$O${}_{7}$, and for potential phases which constitutes the transition pathway from *I4/mmm* → *Cmc2${}_{1}$*. The *Ccce* phase is considered as being a potential intermediate phase which may occur as a discontinuous first order phase transition (represented by the dashed box) and the *Fmm2* structure has very low probability of occurring according to the phonon dispersion relations (represented in faded dark red). The different views of the HfO${}_{6}$ octahedral tilts/rotations are shown for each phase. The red and light green spheres represent the O and Hf-site ions, respectively, and the black arrows indicate the directions of the O rotation and/or tilt around the Hf atom.

The calculated hyperfine parameters, namely the EFG principal component $V_{\mathrm{zz}}$ and the asymmetry parameter η, at Sr${}_{1}$, Sr${}_{2}$ and Hf atoms in the different phases, are shown in Figure 6. From our results the different pathways from the high symmetric phase, *I4/mmm*, and the polar *Cmc2${}_{1}$* structure, can be followed through the $V_{\mathrm{zz}}$ and η parameters. At the Hf site, we observe that the lowest values for $V_{\mathrm{zz}}$ are found in the high-symmetry *I4/mmm* phase, with $V_{\mathrm{zz}}$ = 16.38 V/Å${}^{2}$ and η = 0.00 due to the center-symmetric positions of the nondistorted octahedral HfO${}_{6}$ cages. On the other hand, the largest magnitude of η, at the Hf site, (η = 0.70) was obtained for the *Cmcm* system, which corresponds to the tilting of the cages and the largest contribution to the distortion towards the polar ground-state phase (as observed in Table 3). However, the $V_{\mathrm{zz}}$ = 48.87 V/Å${}^{2}$, obtained for the *Cmcm* phase is lower than that of the *Cmca* phase (associated with the in-plane rotations of the HfO${}_{6}$ perovskite cages as shown in Figure 6) corresponding to the largest magnitude of the principal EFG component, with $V_{\mathrm{zz}}$ = −89.63 V/Å${}^{2}$ and η = 0.13.

The characterization of the Sr local environments may also allow one to probe the HfO${}_{6}$ octahedral rotations that underlie the structural phase transitions of the system. Therefore, with respect to the rock-salt Sr${}_{2}$ sites, for *Fmm2* phase, we find that the value of $V_{\mathrm{zz}}$ and η are very close to that of the high symmetric *I4/mmm* phase, with $V_{\mathrm{zz}}$ = −76.44 V/Å${}^{2}$, η = 0.00 and $V_{\mathrm{zz}}$ = −77.91 V/Å${}^{2}$, η = 0.00 respectively corresponding to the absence of the octahedral rotations and/or tilts of the rock-salt environment. This similarity is understandable as the *Fmm2* phase results from a very small amplitude displacement of the Sr ions. Upon octahedral rotations we observe that the value of η increases significantly in accordance to what was recently observed in Ca${}_{2}$MnO${}_{4}$ where η increases from 0 to 0.8 as the Mn-O-Mn bond angle decreases from 180º to 160º [55]. Moreover, we observe that the asymmetry parameter evidences the largest values with η = 0.77 and η = 0.96, for the *Cmcm* and the ground-state *Cmc2${}_{1}$*, respectively. In these two mentioned phases the lowest values for $V_{\mathrm{zz}}$ are found with $V_{\mathrm{zz}}$ = −54.48 V/Å${}^{2}$ and $V_{\mathrm{zz}}$ = −58.31 V/Å${}^{2}$. Interestingly, we observe that the highest values of $V_{\mathrm{zz}}$ at the rock-salt site are observed for the *Cmca* and *Ccce*, which are the phases which respectively correspond to the in-plane and out-of-plane rotations of the cages (Figure 6). The asymmetry parameters are very close to each other (η = 0.606 and η = 0.672).

The EFG values found here for the *I4/mmm*, *Ccce*, and *Cmc2${}_{1}$* phases at the Sr${}_{2}$ rock-salt site and Sr${}_{1}$ perovskite site show similar trends as those calculated for Ca${}_{3}$Mn${}_{2}$O${}_{7}$ system at analogous sites [18]. In addition, we observe that the largest η values at the perovskite sites, Sr${}_{1}$, are for the ground-state *Cmc2${}_{1}$*, with η = 0.73, and followed by the *Cmcm* phase with η = 0.72. On the other hand, the *Cmca* phase ($X_{2}^{+}$ mode) evidences largest $V_{\mathrm{zz}}$ magnitudes, not only for the Hf sites, as already mentioned, but also for both Sr${}_{1}$ and Sr${}_{2}$ sites, with $V_{\mathrm{zz}}$ = 74.21 V/Å${}^{2}$ and $V_{\mathrm{zz}}$ = −119.55 V/Å${}^{2}$, respectively.

As mentioned before, the *Ccce* phase is not considered to be in the transition pathway from *I4/mmm* to *Cmc2${}_{1}$*, when assuming group theory analysis, as they are not in a group-subgroup relation. Nevertheless, this phase might manifest as an intermediate one, displaying a discontinuous first-order phase transition given the considerable gain of total energy with respect to *I4/mmm*. The EFGs values of the *Ccce* system ($V_{\mathrm{zz}}$ = −58.21 V/Å${}^{2}$, $V_{\mathrm{zz}}$ = −90.77 V/Å${}^{2}$ and $V_{\mathrm{zz}}$ = 72.37 V/Å${}^{2}$ for the Sr${}_{1}$, Sr${}_{2}$ and Hf sites, respectively) are larger than the ones found for *Cmc2${}_{1}$*, *Cmcm*, *Fmm2*, and *I4/mmm* phases, although with lower value when compared to the *Cmca* phase. Moreover, the asymmetry parameter, η, for the rock-salt Sr${}_{2}$-site is also quite high, with η = 0.67; however, for the remaining two sites η approximates to zero. These results are in agreement with the experimental data in Reference [18] for the Ca${}_{3}$Mn${}_{2}$O${}_{7}$ compound, in which the highest magnitudes of $V_{\mathrm{zz}}$ were observed for the *Ccce* phase when compared to the ground-state structural or *I4/mmm* phase.

In a more comprehensive point of view, and considering a close inspection of the EFG behavior at each site (perovskite Sr${}_{1}$, rock-salt Sr${}_{2}$ and Hf sites) across the potential phases, which constitutes the transition pathway from *I4/mmm* → *Cmc2${}_{1}$*, clear EFG signatures can be observed. For example, for the rock-salt site the *I4/mmm* → *Cmca* → *Cmc2${}_{1}$* pathway has the following EFG signature: $V_{\mathrm{zz}}$ = −77.91 V/Å${}^{2}$ η = 0.00 → $V_{\mathrm{zz}}$ = −119.55 V/Å${}^{2}$ η = 0.61 → $V_{\mathrm{zz}}$ = −58.31 V/Å${}^{2}$ η = 0.96, as depicted in Figure 6. In addition, the hypothetical *I4/mmm* → *Ccce* → *Cmc2${}_{1}$* path shows the difference in the intermediate phase with $V_{\mathrm{zz}}$ = −90.77 V/Å${}^{2}$, η = 0.67, with magnitudes of both parameters being quite close magnitudes of the asymmetric parameters. A similar situation is observed for the Sr${}_{1}$ and Hf sites where distinguishable EFG ($V_{\mathrm{zz}}$, η pairs) within each EFG paths are evident (see Figure 6). These results show that an experimental EFG measurement, by using hyperfine techniques such as nuclear quadrupole resonance (NQR) or perturbed angular correlation (PAC), might allow to ascertain for the correct phases/pathways, namely the ones connected by group-subgroup relations. In such a way, these techniques can inform about the subtle octahedral tilting and rotations which are typically not easily accessible by long-range crystallographic techniques. The potential transition pathway, viewed from the rock-salt (Sr${}_{2}$) site, may be correctly studied using PAC, as demonstrated in [18,55]. Moreover, the Hf site may be studied using the ${}^{181}$Hf PAC probe. In addition, and as recently mentioned by Zao et al. [56], a local description of oxides is fundamental in the understanding of their properties, since polymorphs should be present in a given sample. In this sense, hyperfine quantities are especially well suited to probe very local environments.

## 4. Discussion and Conclusions

From symmetry mode analysis and energetic stability we infer that the most probable pathway transition from the aristotype towards the polar ground-state system of SHO is: *I4/mmm* > *Cmcm* > *Cmca* > *Cmc2${}_{1}$*. The energetic trend is here confirmed. Although the energetics of the *Ccce* phase is higher than that of *Fmm2*, we do not consider it to constitute the pathway between *I4/mmm* → *Cmc2${}_{1}$* according to group theory analysis, since the condensation of the $X_{1}^{-}$ mode does not naturally occur as being a continuous second order phase transition for the SHO system. However, there is the possibility of such a mode being induced as a first-order transition (for example, through application of an external perturbation such as pressure), as observed in the literature for other systems. Moreover, the band gap widths also increase in a similar trend according to the energetic stability and shows slight variations along the potential phases that constitute the transition pathway. We must, however, note that the symmetry-breaking is mostly driven by the O octahedral rotations with different symmetry modes, namely the $X_{2}^{+}$ and the $X_{3}^{-}$ distortions, which respectively lower the high-symmetry to the *Cmcm* and *Cmca* phases. These O rotations and tilts are the primary order parameter of the phase transition for which a secondary order parameter will induce the spontaneous polarization of the *Cmc2${}_{1}$* system. The combination of both the Q${}_{X_{2}^{+}}$ and Q${}_{X_{3}^{-}}$ modes lower the overall energy of the system and the polarization arises due to the coupling of a hybrid order parameter (tilts and rotations of the oxygen octahedral cages and the Sr displacements). Based on the phonon dispersions, we may infer that the *Fmm2* may not exist, since we observe that the phonon branches are stable at the Γ-point for the *I4/mmm* structure, implying that the system will most likely not undergo a transition to this phase. In addition, by observing Figure 3 it is here shown that the $\Gamma_{5}^{-}$ mode alone increases the total energy, further evidencing that the *I4/mmm* → *Fmm2* transition will most likely not occur. The *I4/mmm* instability at the **X**-point will drive the transition directly to the *Cmcm* or *Cmca* space groups, from which the Γ-point imaginary modes of either these two phases will probably direct the final transition to *Cmc2${}_{1}$*. We observe that the magnitudes of $V_{\mathrm{zz}}$ and η are extremely sensitive to the octahedral rotations and tilting distortions across the phase transition path. Clearly distinct EFG signatures are observed at each (perovskite Sr${}_{1}$, rock-salt Sr${}_{2}$ and Hf) site for the potential phase that constitutes the transition pathway from *I4/mmm* → *Cmc2${}_{1}$*. These EFG results are particularly relevant to establish experimentally the theoretical predicted phases/pathways using hyperfine measurements, especially when crystallographic long-range methods fail. We hope that the results presented here will stimulate further measurements into this challenging system.

## Figures and Tables

**Table 1:** Lattice parameters and equilibrium volume (per formula unit) of SHO for the *I4/mmm*, *Ccce*, *Fmm2*, *Cmcm*, *Cmca*, *Cmc2${}_{1}$* structural phases obtained by using the PBEsol functional. Values in brackets were obtained with QE, whereas all remaining were obtained with WIEN2k.

Space Group	a${}_{0}$, b${}_{0}$, c${}_{0}$ (Å)	V${}_{0}$ (Å${}^{3}$)
*I4/mmm*	4.103, 4.103, 20.889	175.83
	[4.058, 4.058, 20.685]	[170.31]
*Ccce*	5.694, 5.692, 21.609	175.09
	[5.663, 5.664, 21.050]	[168.80]
*Fmm2*	5.794, 5.792, 20.892	175.28
	[5.738, 5.738, 20.688]	[170.28]
*Cmcm*	5.863, 5.752, 20.775	175.15
	[5.780, 5.705, 20.517]	[169.14]
*Cmca*	5.777, 5.777, 20.798	173.53
	[5.669, 5.670, 21.039]	[169.07]
*Cmc2${}_{1}$*	5.795, 5.742, 20.973	174.47
	[5.725, 5.713, 20.677]	[169.07]

**Table 2:** Summary of the basis modes in the distortion of SHO, from the *I4/mmm* to the *Cmc2${}_{1}$* phase, distributed per type of Wyckoff position (WP). Numbers in parenthesis indicate the number of modes for each irreducible representations (IR).

Atoms	WP	Modes
Sr${}_{1}$	2b	$\Gamma_{5}^{-}$(1), $X_{3}^{-}$(1)
Sr${}_{2}$	4e	$\Gamma_{1}^{+}$(1), $\Gamma_{5}^{-}$(1), $X_{3}^{-}$(1)
Hf	4e	$\Gamma_{1}^{+}$(1), $\Gamma_{5}^{-}$(1), $X_{3}^{-}$(1)
O${}_{1}$	2a	$\Gamma_{5}^{-}$(1), $X_{3}^{-}$(1)
O${}_{2}$	8g	$\Gamma_{1}^{+}$(1), $\Gamma_{5}^{-}$(2), $X_{2}^{+}$(2), $X_{3}^{-}$(1)
O${}_{3}$	4e	$\Gamma_{1}^{+}$(1), $\Gamma_{5}^{-}$(1), $X_{3}^{-}$(1)

**Table 3:** Mode decomposition indicating the amplitudes (Å) of each IR distortion component. From left to right, the table lists (1) the k wave-vector for each IR present in the distortion; (2) the restricted direction of the IR space; (3) the isotropy subgroup; (4) the amplitudes of the symmetry components. Amplitudes are normalized with respect to the primitive unit-cell of the high-symmetry structure.

k-Vector	IR	Subgroup Dimension	Amplitude (Q${}_{\mathrm{IR}}$)
(0,0,0)	$\Gamma_{1}^{+}$	*I4/mmm* (139)	0.0856
(0,0,0)	$\Gamma_{5}^{-}$	*Fmm2* (42)	0.2953
(1/2,1/2,0)	$X_{2}^{+}$	*Cmca* (64)	0.5487
(1/2,1/2,0)	$X_{3}^{-}$	*Cmcm* (63)	0.7838

**Table 4:** Relative energy difference of the potential phases of SHO with respect to the ground-state *Cmc2${}_{1}$* system.

Space Group	Δ E (eV)
*I4/mmm* (139)	1.946
*Ccce* (68)	0.381
*Fmm2* (42)	0.354
*Cmcm* (63)	0.101
*Cmca* (64)	0.026
*Cmc2${}_{1}$* (36)	0.000

**Table 5:** Energy band gap widths for different structural phases.

Space Group	PBEsol E${}_{g}$ (eV)	mBJ E${}_{g}$ (eV)
*I4/mmm* (139)	3.62	5.22
*Ccce* (68)	4.19	5.86
*Fmm2* (42)	3.63	5.23
*Cmcm* (63)	3.95	5.67
*Cmca* (64)	4.24	5.93
*Cmc2${}_{1}$* (36)	4.25	5.95

## Data Availability

The data that supports the work presented within this paper is available by the corresponding author upon reasonable request.
